# Dr. Phineas Sewall Stearns

**Published:** 1913-08

**Authors:** 


### OBITUARIES.

---

*Readers are requested to report promptly the death of any physician in Western New York, or former residents of this region, or graduates of any medical school in Western New York and to call the attention of the families of the deceased, our desire to publish adequate obituary notices.*

---

Dr. Phineas Sewall Stearns, P. & S., Boston, 1882, formerly in practice at Owego and later at Buffalo, died in Stony Creek. N. Y., May 5, aged 83.

